# Changes in Mental Health among Psychiatric Patients during the COVID-19 Pandemic in Hong Kong—A Cross-Sectional Study

**DOI:** 10.3390/ijerph19031181

**Published:** 2022-01-21

**Authors:** Joyce Tik-Sze Li, Chui-Ping Lee, Wai-Kwong Tang

**Affiliations:** 1 School of Pharmacy, Faculty of Medicine, The Chinese University of Hong Kong, Hong Kong SAR, China; joycetsli@link.cuhk.edu.hk; 2 Department of Psychiatry, Faculty of Medicine, The Chinese University of Hong Kong, Hong Kong SAR, China; tangwk@cuhk.edu.hk

**Keywords:** COVID-19, psychiatric, mental health, depression, anxiety, stress

## Abstract

Background: The COVID-19 pandemic has had substantial impacts on citizens’ daily living. Concerns over mental health issues are rising. Recent studies assessing the psychosocial impact of COVID-19 on the general public revealed alarming results. Meanwhile, the impact of the COVID-19 pandemic on mental health among patients with pre-existing psychiatric disorders remained unclear. Methods: Patients diagnosed with anxiety disorders, depressive disorders, bipolar disorders, or schizophrenia were invited to complete a survey between July and October 2020. The survey collected information on subjects’ demographics, accommodation status, changes in mental health status during the COVID-19 outbreak, and the factors that affect subjects’ mental health during COVID-19. The primary outcome of this study was the change in mental health, defined by psychiatric symptom change and patient satisfaction on symptom control. The secondary outcomes were patients’ emotional status—measured by the Depression, Anxiety and Stress Scale (DASS-21)—during the COVID-19 pandemic and factors that impacted patients’ mental health during the COVID-19 pandemic. Results: Out of the 294 patients recruited, 65.0% were living in hostel while 35.0% were living in the community. The proportion of patients with ‘unsatisfied’ or ‘very unsatisfied’ mental disease control increased from 10.2% to 17.1% after the COVID-19 outbreak (*p* < 0.001). Under the DASS-21 questionnaire, 24.2% subjects, 32.6% subjects, and 18.9% subjects were classified as severe or extremely severe in terms of the level of depression, anxiety, and stress they experienced, respectively. Patients living in the community, patients with mood disorders, and female patients reported significantly worse control over anxiety and mood symptoms. The three major factors that affected patients’ mental health during COVID-19 were ‘reduced social activities’, ‘worries over people around getting infected’, and ‘reduced exercise’. Conclusion: Psychiatric patients in general have poorer disease control after the COVID-19 outbreak. Patients in the community appeared to be more affected than patients residing in hostels. More efforts should be directed to screening patients with pre-existing mental health disorders to enable timely interventions.

## 1. Introduction

The Coronavirus Disease 2019 (COVID-19) is an infectious disease caused by the severe acute respiratory syndrome coronavirus 2 (SARS-CoV-2) [1]. Common symptoms of COVID-19 include fever, dry cough, and fatigue [2]. Other symptoms include loss of taste or smell, nasal congestion, conjunctivitis, sore throat, headache, muscle ache, joint pain, and diarrhoea [2]. The virus, SARS-CoV-2, was first identified in Wuhan, the capital city of Hubei, China [3]. It then quickly spread globally, and the World Health Organization (WHO) declared COVID-19 a pandemic on 11 March 2020 [4]. Until 10 March 2021, 117,356,130 confirmed cases and 2,609,330 deaths had been reported globally [5].

COVID-19 is not only a global challenge to citizens’ physical health, but also their mental and social wellbeing [6,7,8]. Government authorities have enforced various pandemic prevention measures, including mandatory health quarantine, suspension of classes, prohibition of group gathering, and compulsory COVID-19 testing [9]. Citizens are also advised to minimize social activities, which implies huge changes from their usual lifestyle. In addition, many people were panicking due to shortage of supplies, such as face masks, hand sanitizer, soap, and bleach, especially in the first quarter of 2020 [10]. There was news reporting on crowds lining up overnight outside pharmacies to buy face masks and alcohol swaps, supermarkets with most of the goods sold out, and people crying due to inadequate face masks [11,12,13]. It is obvious that COVID-19 has affected many aspects of citizens’ daily life.

Apart from COVID-19, other major infectious disease outbreaks in the 21st century included the severe acute respiratory syndrome (SARS) in 2003, the H1N1 influenza in 2009, the Middle East respiratory syndrome (MERS) since 2012, and the Ebola virus disease between 2014 and 2016 [14,15]. Previous studies had revealed that infectious disease outbreak could have long-lasting impacts on people’s mental health [16,17]. For example, the 2003 SARS outbreak in Hong Kong was associated with a higher suicide rate among older adults [18]. Concerns over mental health issues were brought up quickly since the COVID-19 outbreak. Studies had attempted to assess the immediate psychological impact of COVID-19 in different countries [19,20,21,22]. A cross-sectional study surveyed 1210 adults from 194 cities in China and found that 53.8% of respondents rated the psychological impact of the outbreak as moderate or severe [23]. There were 16.5% who reported moderate to severe depressive symptoms, 28.8% who reported moderate to severe anxiety symptoms, and 8.1% who reported moderate to severe stress levels. A study that surveyed 2458 respondents in Denmark between March and April 2020 found a mean score of 62.0 using five-item WHO-5 well-being scale, which was significantly lower than the 64.3 reported in a similar study in 2016, indicating a poorer subjective psychological well-being in 2020 [24]. A cross-sectional study surveying 3480 Spanish in March 2020 revealed that 18.7% of the sample showed depressive, 21.6% showed anxiety and 15.8% showed post-traumatic stress disorder symptoms [25].

The psychological impact of COVID-19 might be more pronounced in patients with mental disorders [26]. Patients with mental disorders might be more susceptible to emotional stress brought on by the pandemic, resulting in relapses or worsening of their pre-existing mental health condition [27,28]. In addition, mental health services that patients could enjoy were limited by COVID-19 [29,30]. Manpower and healthcare resources were deployed to fight the virus. Some patients had their follow-up period lengthened to reduce pathogen exposure in institutions [31]. Community centres were also temporary closed in accordance with social distancing policies [32]. A survey conducted by WHO on 130 countries revealed that although the demand was rising, critical mental health services were disrupted or halted in 93% of countries worldwide [33]. Thus, patients indeed receive less support after the COVID-19 outbreak despite apparent needs.

Preliminary data had suggested that psychiatric patients experienced more severe negative psychological impact from COVID-19 [26,34,35,36]. A case-control study conducted by Fengyi et al. revealed that psychiatric patients in China had higher mean Impact of Event Scale-Revised (IES-R); Depression, Anxiety and Stress Scale (DASS-21); and Insomnia Severity Index (ISI) scores than healthy controls during the early stage of the COVID-19 outbreak [37]. Recent studies targeting psychiatric patients with different background—such as age groups, occupations, and nationalities—revealed high proportion of patients suffering from depression, anxiety, and acute stress [38,39,40]. Patients also reported reduced quality of life in some studies [41,42]. On the other hand, reported changes in patients’ psychiatric symptoms control varied among studies [41,43]. For example, in a study by Favreau et al., more than 50% of recruited inpatients with mental disorders in Germany reported a general worsening of their symptomatology [41]. In the study by Berardelli et al., psychiatric patients admitted to a public psychiatric clinic reported more frequent suicide attempts, but not suicide ideation, during the COVID-19 pandemic than before [43]. The variation in patients’ responses could be attributed by multiple factors—such as the severity, time of outbreak, and government measures against COVID-19, which differed across countries. Nevertheless, the major factors that affected patients’ mental health were not investigated in most studies. In addition, most of the published data were conducted on inpatients, patients attending psychiatric clinics, or patients living in rehabilitation communities. Patients who lived in the community and received less support or who did not seek help proactively from health authorities might be underrepresented.

Despite being one of the regions with COVID-19 cases reported in the early stage, data on the impact of COVID-19 on psychiatric patients in Hong Kong remain scarce. While Hong Kong is well known for its extremely low average living area per capita, it would be interesting to study how the strict quarantine measures, extensive work-from-home arrangement, and lack of preventive supplies may have affected psychiatric patients with different accommodation status—i.e., residing in the community vs. hostel—as they received different levels of support. Overall, more data on the changes in mental health among Hong Kong psychiatric patients before and after COVID-19 are needed.

The current study aimed to investigate the changes in mental health among psychiatric patients during the COVID-19 pandemic in Hong Kong. The major objectives were to investigate the change in satisfactory level on psychiatric disease control, the change in psychiatric symptom control, and the emotional status among psychiatric patients during the COVID-19 pandemic, as well as to identify factors that impacted patients’ mental health during the COVID-19 outbreak.

## 2. Materials and Methods

### 2.1. Study Design and Study Population

The current study was a cross-sectional study conducted between July and October 2020. Subjects were recruited through local patient groups which provided support to psychiatric patients living in the community and hostels. A total of 10 service sites for community and hostel patients were involved. Snowball sampling was also adopted. Patients who were included were those who aged 18 years old or above, were diagnosed with anxiety disorders, depressive disorders, bipolar disorders, or schizophrenia, and were taking at least 1 medication indicated for mental disorders. Patients were asked to indicate their psychiatric diagnoses and current medications on the questionnaires. Patients who could not read or understand Chinese or were unable to provide informed consent were excluded.

Patients were invited to complete a questionnaire either in paper form or through an online link. The survey was set to determine the changes in disease control among psychiatric patients, and to identify factors which had greater impact on patients’ mental health during the COVID-19 outbreak. The questionnaire consisted of four parts.

In the first part, subjects’ demographic data—which included their age, gender, education level, type of mental disorders, year of diagnosis, number of current psychiatric medications, whether the subject had been put on mandatory health quarantine, and whether the subject had been diagnosed with COVID-19—were collected.

In the second part, subjects completed the Depression, Anxiety and Stress Scale (DASS-21) which assesses their current emotional status [44]. DASS-21 assesses subjects’ depression, anxiety, and stress levels based on 21 questions. It had been validated in multiple studies and demonstrated high internal consistency [45]. The Chinese version of DASS-21 was available, and DASS-21 had been demonstrated to be reliable and valid in assessing mental health in the Chinese population [46]. It had also been adopted in previous studies related to SARS and COVID-19 [23,47].

In the third part, subjects were asked to report the changes in their symptoms and satisfactory level on psychiatric disease control before and after the COVID-19 outbreak. Information collected included the changes in self-perceived disease control, changes in symptoms control, and changes in psychiatric medications.

In the fourth part, factors that imposed impact on subjects’ mental health during COVID-19 were identified. Some examples were listed out and subjects were asked to rate—using Likert scale of 1 (lowest)–5 (highest)—the extent to which those factors affected their mental health. Examples of the possible factors included overwhelming news, fake news, worries of getting infected, inadequate knowledge on prevention, social distancing, inability to purchase daily necessities, reduced income, and mandatory health quarantine. The possible factors were cited from literature and news reports [6,11,12,13,19].

Written informed consent was obtained from all subjects before they completed the questionnaire.

### 2.2. Outcome Measures

The primary outcome of this study was the change in mental health, defined by psychiatric symptom change and patient satisfaction on symptom control, among psychiatric patients before and after the COVID-19 outbreak. The secondary outcomes were patients’ emotional status, as measured by DASS-21(Moussa, Lovibond, & Laube, 2001, Sydney, Australia), during the COVID-19 pandemic and factors that impacted patients’ mental health.

### 2.3. Data Analysis

Descriptive statistics were used for result analysis. Mean and standard deviation were used for continuous variables, while frequency and percentage were used for categorical variables. Wilcoxon signed rank test was used to compare subjects’ satisfactory level on their mental health before and after the COVID-19 outbreak. Mann–Whitney U-test, Kruskal–Wallis test, logistic regression, and multiple linear regression were used to compare the disease control and DASS-21 scores between subjects with different accommodation statuses, psychiatric history, genders, and age groups. Chi-square testing was used to compare any difference in nominal outcome variables. A *p*-value of less than 0.05 was considered statistically significant. All statistical analyses were performed using IBM SPSS Statistics v26 (IBM SPSS Statistics for Windows, Version 26.0. Armonk, NY, USA: IBM Corp).

## 3. Results

### 3.1. Subject Demographics

A total of 313 responses, 197 in paper form and 116 through the online link, were collected between July and October 2020. After screening against the inclusion and exclusion criteria, 294 subjects were included in the analysis. Responders who claimed that they were not diagnosed with anxiety disorders, depressive disorders, bipolar disorders, or schizophrenia, or were not taking any medication indicated for mental disorders, were excluded. Among the 294 subjects, 191 (65.0%) were hostel residents while 103 (35.0%) were from the community. There were 32 (10.9%) aged 18–29, 123 (42.0%) aged 30–44, 107 (36.5%) aged 45–59, and 31 (10.6%) aged 60 or above. One hundred and sixty-three (57.2%) subjects were male. There were 173 (58.8%) subjects diagnosed with schizophrenia, 99 (33.7%) with depressive disorders, 56 (19.0%) with anxiety disorders, and 48 (16.3%) with bipolar disorders. A total of 124 subjects (42.2%) had either depressive disorders or anxiety disorders (the D + A subgroup) while 185 subjects (62.9%) had either bipolar disorders or schizophrenia (the B + S subgroup). In the hostel subgroup, there was a higher proportion of subjects with schizophrenia (70.7% in hostel vs. 36.9% in community) or taking anti-psychotics (67.2% vs. 34.0%). In the community subgroup, a higher proportion of subjects with anxiety disorders (34.0% in community vs. 11.0% in hostel), depressive disorders (55.3% vs. 22.0%), taking anti-depressants (67.0% vs. 39.0%), or taking sedatives/hypnotics (42.3% vs. 27.1%) were reported. A total of 35 (12.0%) subjects were newly diagnosed with mental illnesses during the COVID-19 outbreak. Subjects’ demographic data were shown in Table 1.

### 3.2. Changes in Patient Satisfaction on Mental Disease Control and Self-Perceived Symptom Change

A total of 30 (10.2%) subjects rated their mental disease control as ‘unsatisfied’ or ‘very unsatisfied’ before COVID-19. The number increased to 50 (17.1%) after the COVID-19 outbreak (*p* < 0.001). In the community subgroup, the number of subjects who had ‘unsatisfied’ or ‘very unsatisfied’ mental disease control increased from 12 (11.7%) to 32 (31.1%) after the COVID-19 outbreak (*p* < 0.001). The difference was not significant in the hostel subgroup. Subjects from both the D + A subgroup and the B + S subgroup reported significantly lower satisfaction levels on mental disease control after the COVID-19 outbreak.

Regarding specific symptom control, 149 (53.6%) subjects reported worsened control over mood symptoms (anxiety, mania, depression, panic attack, insomnia, loss of interests, poor concentration, poor appetite, or suicidal ideation) while 39 (18.6%) subjects reported worsened control over psychotic symptoms (hallucination or delusion). Logistic regression showed that female (*p* < 0.001), community subgroup (*p* < 0.001), and patients with depressive disorders (*p* = 0.019) or anxiety disorders (*p* = 0.014) were associated with poorer control over mood symptoms. No correlation between psychotic symptoms control and subjects’ demographics was observed.

There were 82 (28.5%) subjects who had their psychiatric medication changed after the COVID-19 outbreak. The majority of them had their dose increased or number of medications increased (55 out of 82; 67.1%). In the community subgroup, 30 (29.1%) had medication changes, in which 21 (70.0%) had dose increased or number of medications increased. As for the hostel subgroup, 52 (28.1%) had medication changes, in which 34 (65.4%) had their dose or number of medications increased. When asked how they expected their mental health would change if the COVID-19 pandemic persisted for half-a-year or longer, a total of 100 subjects (34.2%) expected that their mental health would be ‘slightly worsen’ or ‘greatly worsen’ (*p* < 0.001). The numbers were significantly higher in the community subgroup (61.1% in community vs. 19.6% in hostel; *p* < 0.001).

### 3.3. DASS-21: Assessment of Subjects’ Depression, Anxiety, and Stress Levels

Table 2 summarised subjects’ DASS-21 scores. Overall, 69 (24.2%) subjects, 93 (32.6%) subjects, and 54 (18.9%) subjects were classified as severe or extremely severe in terms of level of depression (DASS-21 depression score ≥21), anxiety (DASS-21 anxiety score ≥15), and stress (DASS-21 stress score ≥26), respectively. The mean DASS-21 depression score, anxiety score, and stress score were 12.31 ± 11.43 (mild), 11.24 ± 9.94 (moderate), and 13.80 ± 11.25 (normal), respectively. Multiple linear regression showed that subjects from the community subgroup, the D + A subgroup, and female subjects got significantly higher mean DASS-Depression (adjusted R^2^ = 0.119), DASS-Anxiety (adjusted R^2^ = 0.102), and DASS-Stress (adjusted R^2^ = 0.185) scores. No significant difference in mean DASS-21 scores among subjects with different age or education level was observed.

There were 106 (36.1%) subjects who were classified as having severe or extremely severe levels of depression, anxiety, or stress. The proportion was significantly higher in the community subgroup and the D + A subgroup. There were 36 (35.0%) subjects, 49 (47.6%) subjects, and 37 (35.9%) subjects in the community subgroup being classified as severe or extremely severe in terms of level of depression, anxiety, and stress, respectively, compared with 33 (18.1%; *p* < 0.001), 44 (24.2%; *p* = 0.001), and 17 (9.3%; *p* < 0.001) in the hostel subgroup. As for the D + A subgroup, 34 (27.9%), 53 (43.4%), and 42 (34.4%) were classified as severe or extremely severe in terms of level of depression, anxiety, and stress, respectively, compared with 22 (12.3%; *p* < 0.001), 44 (24.8%; *p* < 0.001), and 32 (18.0%; *p* < 0.001) in the hostel subgroup.

### 3.4. Factors Affecting Mental Health

**Figure 1:**
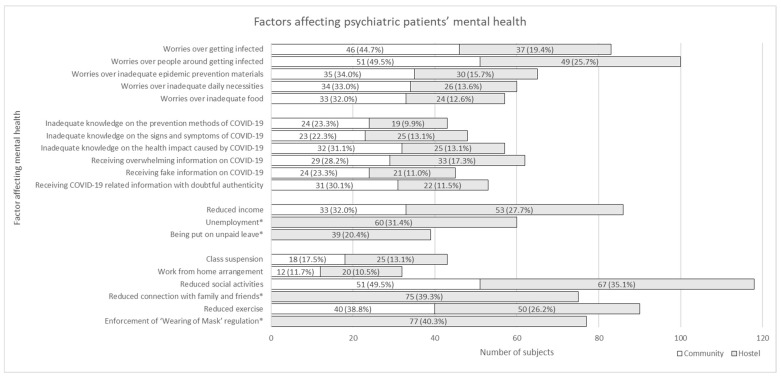
Factors affecting psychiatric patients’ mental health. Note: The numbers here referred to the number of subjects choosing 4 or 5 using Likert scale of 1–5. * These items were only asked in hostel patients.

Figure 1 illustrated the extent to which different factors impacted subjects’ mental health. There were 118 (40.1%) subjects who rated 4 or above for ‘reduced social activities’ using Likert scale of 1–5, followed by ‘worries over people around getting infected’ (100 out of 294, 34.0%) and ‘reduced exercise’ (90 out of 294, 30.6%). In the community subgroup, the top three factors were ‘reduced social activities’ (51 out of 103, 49.5%), ‘worries over people around getting infected’ (51 out of 103, 49.5%), and ‘worries over getting infected’ (46 out of 103, 44.7%). As for the hostel subgroup, the top three factors were ‘enforcement of “Wearing of Mask” regulation’ (77 out of 191, 40.3%), ‘reduced connection with family and friends’ (75 out of 191, 39.3%), and ‘reduced social activities’ (67 out of 191, 35.1%). Responses from the D + A subgroup and the B + S subgroup were similar. The top three factors in both subgroups were ‘reduced social activities’, ‘worries over people around getting infected’, and ‘reduced exercise’.

**Figure 2:**
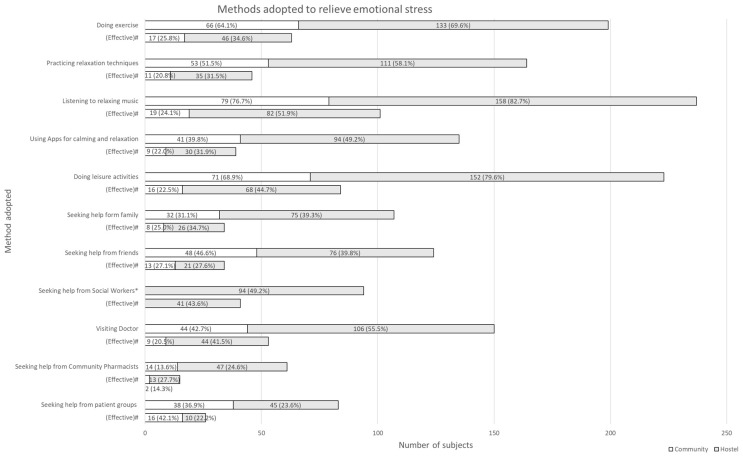
Methods that patients adopted to relieve emotional stress. Note: * This item was only asked in hostel patients. # The percentages referred to the proportion of subjects who had adopted the methods listed and found them effective.

Figure 2 summarised the methods that subjects adopted to relieve their emotional stress. Most subjects had tried ‘listening to relaxing music’ (237 out of 294, 80.6%), followed by ‘doing leisure activities’ (223 out of 294, 75.9%), and ‘doing exercise’ (199 out of 294, 67.7%). Methods adopted by subjects with different accommodation status or psychiatric history were similar. Regarding the channels for subjects to seek help on mental health issues, highest number of subjects sought help from doctor (150 out of 294, 51.0%), followed by friends (124 out of 294, 42.2%), and family (107 out of 294, 36.4%). A total of 89 subjects (30.3%) indicated that they did not seek help from any parties listed on the questionnaire. Out of the 89 subjects, 22 (24.7%) were classified as having severe or extremely severe level of depression, anxiety, or stress based on DASS-21 scores. No correlation between the methods adopted or the perceived effectiveness of methods chosen and subjects’ satisfaction level on psychiatric disease control or DASS-21 scores was observed.

## 4. Discussion

Results from the current study were in line with recent studies which revealed worsened symptoms or disease control in psychiatric patients since the COVID-19 outbreak. Higher proportion of patients were unsatisfied with their mental health after COVID-19. Community patients reported worsened control over a range of anxiety and mood symptoms and they appear to be more affected than their hostel counterparts.

Until March 2021, COVID-19 has struck Hong Kong for over one year. The outbreak pattern could be described as four distinct waves [48]. The first and the second waves occurred in the first half of 2020. There were around 1200 cases, in which most of them were imported cases [48]. The third wave happened between July and October 2020. Over 3000 confirmed cases were reported, and majority were locally infected cases [49]. The fourth wave began in October 2020 and continued till March 2021. The current study was conducted between July and October 2020, which reflected subjects’ mental status during the third wave of outbreak.

While the impact of SARS-CoV-2 on physical health and mortality might be of greatest interest during the initial outbreak, issues with mental health had also attracted attention quickly [50,51]. A 16% increase in number of local citizens calling the 24-h community mental health support hotline was reported within the first four months of the COVID-19 outbreak [52]. A population-based study surveying 500 local citizens between April and May 2020 revealed that 19% of respondents had depression and 14% had anxiety [53]. Government authorities and non-government organizations had published educational materials to remind citizens to be aware of emotional crises, introduce relaxation techniques, and encourage citizens to seek help when necessary [54,55,56].

In the current study, 24.2%, 32.6%, and 18.9% subjects had severe or extremely severe level of depression, anxiety, and stress, respectively. These proportions were higher than those reported in China. A case-control study revealed that out of the 76 studied psychiatric patients in China, 13.2% had severe or extremely severe depression level, 14.4% had severe or extremely severe anxiety level, and 7.8% had severe or extremely severe stress level based on their DASS-21 scores [37]. Comparing with other overseas studies, it is also clear that the mental status among Hong Kong psychiatric patients is quite alarming. For example, a study surveying 1106 subjects in Saudi Arabia in April 2020 found 16.4% subjects had a severe or extreme DASS-Depression score, 13.9% had a severe or extreme DASS-Anxiety score, and 13.7% had a severe or extreme DASS-Stress score [57]. Another study surveying 1879 subjects in the Philippines between March and April 2020 found 4.2%, 11.1%, and 3.9% had severe or extreme DASS-Depression, Anxiety, and Stress scores, respectively. The percentages were not as high as that reported in the current study [58]. The higher level of depression, anxiety, and stress reported in the current sample may be related to the time of conducting this study. The current study was conducted during the third wave of outbreak in Hong Kong. There was a sudden surge in the number of COVID-19 cases and the number of people being put under mandatory quarantine which might have affected patients’ emotion. The more stringent criteria and longer duration of lockdown might have also contributed to the higher depression, anxiety, and stress levels. In addition, Hong Kong citizens could easily associate COVID-19 with SARS, the viral respiratory disease with 1755 reported cases and 299 mortality cases in Hong Kong in 2003. COVID-19 and SARS shared many similarities. For example, both diseases were caused by coronavirus, both diseases could present with respiratory symptoms, both diseases were first identified in China, and both diseases had caused a long duration of class suspension [59,60]. Hong Kong citizens might be more sensitive to infectious disease outbreak after experiencing SARS.

Results from the current study revealed that COVID-19 had a more profound impact on mental health among patients living in the community than those living in hostel. In terms of disease control, higher proportion of subjects from the community experienced worsen symptom control over mood and anxiety symptoms compared to hostel residents. The difference might be related to their psychiatric history. There was a higher proportion of subjects with anxiety disorders or mood disorders, who reported worse symptom control and higher mean DASS-21 scores, in the community subgroup. These findings were in line with recent studies, which revealed that patients with anxiety disorders or depressive disorders presented higher psychological distress or higher prevalence of depression and anxiety symptoms than schizophrenia patients during COVID-19 lockdown [61,62]. Another possible reason for the difference was that patients living in hostel could receive more support than their community counterpart [63]. A surveyed conducted by WHO revealed a widespread disruption of many kinds of critical mental health services, including emergency interventions, counselling, psychotherapy, critical harm reduction services, maintenance treatment for substance abuse, and school and workplace mental health services [33]. The situation in Hong Kong was similar. Many community centres were temporary closed [32]. Some patients had their psychiatric unit follow-up period extended or their therapy sessions cancelled [64]. Services became less accessible for community patients. On the other hand, hostel residents could seek help from onsite social workers, nurses, or other hostel staff easily. Furthermore, the study results showed that the major factors affecting subjects’ mental health included reduced social activities and reduced exercise. Patients living in hostels could interact with other residents or participate in sports activities offered by hostel staff. These activities might help relieve their subjective feeling of isolation and loneliness.

The top three factors that impacted subjects’ mental health the most were ‘reduced social activities’, ‘worries over people around getting infected’, and ‘reduced exercise’. These findings echoed a previous systematic review which reported consistent evidence linking weak social support and loneliness to poor mental health [65]. On the other hand, information overload or inadequate supplies were not identified as major factors affecting mental health. Information overload had been proposed as a contributing factor to poor mental wellbeing during COVID-19 [66]. A study in China found that a longer exposure to social media during the COVID-19 pandemic could increase the likelihood of having anxiety as measured by the GAD-7 [6]. A previous study in South Korea also found positive correlation between social media exposure and formation of risk perceptions during MERS outbreak [67]. Besides, a recent study investigating Hong Kong citizens’ mental health during early phase of COVID-19 suggested that individuals who were more bothered by having not enough surgical masks were more likely to have depression, anxiety, combined depression and anxiety, and worsened mental health [53]. However, inadequate supplies was not identified as a major stress in this study. One possible reason for the different observations was that the current study was conducted during the third wave, which meant a few months after COVID-19’s first emergence. Supply of face mask and alcohol swabs started to stabilise and become accessible by most people. The government had launched a website to deliver correct information to the public and hosted regular press conference to release latest information [9]. Citizens had more channels to obtain correct information as compared with a few months before. Therefore, findings from this study are likely to reflect the enduring impact of COVID-19 on psychiatric patients, instead of the immediate impact.

### 4.1. Practical Implications

Despite the devastating consequences that COVID-19 caused, it might have provided insight on future mental health service development. Existing data are showing that COVID-19 can have substantial mental health impacts on the public. Meanwhile, the pandemic may have boosted citizens’ awareness on mental health. New services such as psychological counselling via digital applications, online therapy sessions, toll-free mental health helplines, and public education through mass media are being developed [68,69,70]. Despite the availability of vaccines, the pandemic is expected to persist especially with new strains of the virus emerging. With 34.2% of subjects expecting their mental health to be negatively affected if the outbreak continues for six months or longer, it is essential to formulate effective strategies to screen and manage mental disorder cases under the new normal. The current study identified 106 (36.1%) subjects with severe or extremely severe levels of depression, anxiety, or stress based on the DASS-21 questionnaire. Various nation-wide psychiatric surveys could be used for rapid screening of anxiety and depression in primary care to identify citizens with alarming emotional statuses [71]. As patients with mood disorders may be more affected by the pandemic, intervention should be directed to maintain their social wellbeing and level of physical activity, which were the major factors affecting patients’ mental health.

### 4.2. Limitations

There are several limitations in this study. Firstly, the questionnaires were distributed and collected between July and October 2020. The number of confirmed COVID-19 cases in Hong Kong varied between months. Subjects who submitted the questionnaires between mid-July and early-September might showed poorer mental health status due to higher number of COVID-19 cases during that period. Secondly, the impact of residential location on subjects’ mental health was not considered. During the third wave of COVID-19, some districts in Hong Kong reported higher numbers of infected or death cases [50]. Residential location might have impacted subjects’ mental health status. Despite these limitations, this is one of the very few studies that examined the psychological impact of COVID-19 on psychiatric patients.

## 5. Conclusions

The current study shows that psychiatric patients in general have poorer disease control after the COVID-19 outbreak. Patients in the community appeared to be more affected than patients residing in hostels. The majority of patients expect that their mental health conditions will worsen if the pandemic persists. As the COVID-19 pandemic continues, effort should be paid to addressing patients’ needs. More efforts should be directed to screening patients with alarming mental health status to enable timely intervention.

## Figures and Tables

**Table 1:** Subject demographics.

		Total (*n* = 294)	Community (*n* = 103)	Hostel (*n* = 191)
Age (*n* = 293)	18–29	32 (10.9%)	8 (7.8%)	24 (12.6%)
	30–44	123 (42.0%)	37 (35.9%)	86 (45.3%)
	45–59	107 (36.5%)	43 (41.7%)	64 (33.7%)
	60 or above	31 (10.6%)	15 (14.6%)	16 (8.4%)
Gender (*n* = 285)	Male	163 (57.2%)	68 (66.0%)	95 (52.2%)
	Female	122 (42.8%)	35 (34.0%)	87 (47.8%)
Education level (*n* = 285)	Never received educated	2 (0.7%)	1 (1.0%)	1 (0.5%)
	Primary or below	27 (9.4%)	8 (7.8%)	19 (10.4%)
	Secondary	190 (66.7%)	60 (58.3%)	130 (71.4%)
	Post-secondary	31 (10.9%)	11 (10.7%)	20 (11.0%)
	Degree or above	35 (12.3%)	23 (22.3%)	12 (6.6%)
Psychiatric diagnoses (*n* = 294)	Anxiety disorders	56 (19.0%)	35 (34.0%)	21 (11.0%)
	Depressive disorders	99 (33.7%)	57 (55.3%)	42 (22.0%)
	Bipolar disorders	48 (16.3%)	19 (18.4%)	29 (15.2%)
	Schizophrenia	173 (58.8%)	38 (36.9)	135 (70.7%)
Duration of psychiatric illness (*n* = 244)	5 years or less	65 (26.6%)	22 (21.8%)	43 (30.1%)
	6–10 years	63 (25.8%)	21 (20.8%)	42 (29.4%)
	11–20 years	64 (26.2%)	36 (35.6%)	28 (19.6%)
	More than 20 years	52 (21.3%)	22 (21.8%)	30 (21.0%)
Newly diagnosed with mental illness during the COVID-19 outbreak (*n* = 292)	Yes	35 (12.0%)	15 (14.6%)	20 (10.6%)
	No	257 (88.0%)	88 (85.4%)	169 (89.4%)
Current psychiatric medications (*n* = 274)	Sedatives/hypnotics	89 (32.5%)	41 (42.3%)	48 (27.1%)
	Anti-depressants	134 (48.9%)	65 (67.0%)	69 (39.0%)
	Anti-psychotics	152 (55.5%)	33 (34.0%)	119 (67.2%)
	Mood stabilizers	104 (38.0%)	31 (32.0%)	73 (41.2%)
Number of psychiatric medications (*n* = 242)	1–2	151 (62.4%)	49 (52.7%)	102 (68.5%)
	3–4	64 (26.4%)	31 (32.0%)	33 (22.1%)
	5–6	20 (8.3%)	10 (10.8%)	10 (6.7%)
	7 or above	7 (2.9%)	3 (3.2%)	4 (2.7%)
Had undergone mandatory health quarantine (*n* = 294)	Yes	10 (3.4%)	0 (0.0%)	10 (5.2%)
	No	284 (96.6%)	103 (100.0%)	181 (94.8%)
Had been diagnosed with COVID-19 (*n* = 292)	Yes	0 (0%)	0 (0.0%)	0 (0.0%)
	No	292 (100%)	103 (100.0%)	189 (100.0%)

**Table 2:** Patients’ emotional status amid COVID-19 pandemic as measured by DASS-21.

	Normal	Mild	Moderate	Severe	Extremely Severe	Mean Score	*p*-Value		Mean Score	*p*-Value
DASS 21-Depression										
Total	142 (49.8%)	32 (11.2%)	42 (14.7%)	31 (10.9%)	38 (13.3%)	12.31 ± 11.43		Total	12.31 ± 11.43	
Community	35 (34.0%)	11 (10.7%)	21 (20.4%)	11 (10.7%)	25 (24.3%)	17.0 ± 12.7	*p* = 0.002	D + A	15.7 ± 11.7	*p* = 0.024
Hostel	107 (58.8%)	21 (11.5%)	21 (11.5%)	20 (11.0%)	13 (7.1%)	9.7 ± 9.7		B + S	10.1 ± 10.6	
DASS 21-Anxiety										
Total	127 (44.6%)	22 (7.7%)	43 (15.1%)	30 (10.5%)	63 (22.1%)	11.24 ± 9.94		Total	11.24 ± 9.94	
Community	32 (31.1%)	8 (7.77%)	14 (13.6%)	16 (15.5%)	33 (32.0%)	14.9 ± 10.9	*p* = 0.001	D + A	14.3 ± 10.6	*p* = 0.006
Hostel	95 (52.2%)	14 (7.7%)	29 (15.9%)	14 (7.7%)	30 (16.5%)	9.2 ± 8.7		B + S	9.1 ± 8.7	
DASS 21-Stress										
Total	169 (59.3%)	26 (9.1%)	36 (12.6%)	36 (12.6%)	18 (6.3%)	13.80 ± 11.25		Total	13.80 ± 11.25	
Community	42 (40.8%)	9 (8.7%)	15 (14.6%)	23 (22.3%)	14 (13.6%)	19.4 ± 11.8	*p* < 0.001	D + A	17.8 ± 10.8	*p* = 0.005
Hostel	127 (69.8%)	17 (9.3%)	21 (11.5%)	13 (7.1%)	4 (2.2%)	10.6 ± 9.6		B + S	11.1 ± 10.6	

Note: There were nine subjects who did not complete the whole set of 21 questions, thus were excluded from the analysis in this part. D + A = subjects with either depressive disorders or anxiety disorders; B + S = subjects with either bipolar disorders or schizophrenia.

## Data Availability

The datasets used and/or analysed during the current study are available from the corresponding author on reasonable request.
